# Effect of Polyunsaturated Fatty Acids on Temozolomide Drug-Sensitive and Drug-Resistant Glioblastoma Cells

**DOI:** 10.3390/biomedicines11030779

**Published:** 2023-03-04

**Authors:** Janaína Alessandra Silva, Alison Colquhoun

**Affiliations:** Department of Cell and Developmental Biology, Institute of Biomedical Sciences, University of São Paulo, São Paulo 05508-000, Brazil

**Keywords:** glioblastoma, polyunsaturated fatty acids, ABC transporters, multiple drug resistance, temozolomide

## Abstract

Glioblastomas (GBMs) are notoriously difficult to treat, and the development of multiple drug resistance (MDR) is common during the course of the disease. The polyunsaturated fatty acids (PUFAs) gamma-linolenic acid (GLA), eicosapentaenoic acid (EPA), and docosahexaenoic acid (DHA) have been reported to improve MDR in several tumors including breast, bladder, and leukaemia. However, the effects of PUFAs on GBM cell MDR are poorly understood. The present study investigated the effects of PUFAs on cellular responses to temozolomide (TMZ) in U87MG cells and the TMZ-resistant (TMZR) cells derived from U87MG. Cells were treated with PUFAs in the absence or presence of TMZ and dose–response, viable cell counting, gene expression, Western blotting, flow cytometry, gas chromatography-mass spectrometry (GCMS), and drug efflux studies were performed. The development of TMZ resistance caused an increase in ABC transporter ABCB1 and ABCC1 expression. GLA-, EPA-, and DHA-treated cells had altered fatty acid composition and accumulated lipid droplets in the cytoplasm. The most significant reduction in cell growth was seen for the U87MG and TMZR cells in the presence of EPA. GLA and EPA caused more significant effects on ABC transporter expression than DHA. GLA and EPA in combination with TMZ caused significant reductions in rhodamine 123 efflux from U87MG cells but not from TMZR cells. Overall, these findings support the notion that PUFAs can modulate ABC transporters in GBM cells.

## 1. Introduction

Glioblastoma (GBM) represents approximately 45.6% of primary malignant brain tumors [1] and is characterized by its rapid and invasive growth pattern [2]. The standard treatment for GBM consists of maximal surgical resection, followed by radiotherapy and chemotherapy with temozolomide (TMZ) [3]. However, despite multiple treatment interventions the prognosis for patients is still extremely poor, with an average survival of only 20 weeks for surgical resection and 36 weeks for surgery and radiotherapy, increasing to 50 weeks with chemotherapy [4].

GBMs usually contain a mixed population of malignant cells, some of which are drug-sensitive while others are drug-resistant. This drug resistance can be characterized as intrinsic, when cells exhibit resistance to chemotherapy from their first exposure to a drug, or acquired, when resistance to chemotherapy occurs during treatment following initially successful chemotherapy [5]. Drug resistance is a result of numerous genetic and epigenetic changes in tumor cells. During treatment, tumor cells can develop resistance to various chemotherapeutic agents, and this is one of the main complications in clinical oncology [5,6]. In GBM, glioma stem cells are thought to be responsible for tumor recurrence and repopulation with a heterogenous and drug-resistant phenotype [5,7]. Two of the main mechanisms of resistance acquired by tumor cells are improved DNA damage repair and increased drug efflux through multidrug resistance membrane proteins (MRPs). Both mechanisms result in reducing the action of chemotherapies such as TMZ in the intracellular environment. These phenomena in GBM are mainly related to increased expression of DNA repair genes such as O-6-methylguanine-DNA-methyltransferase (MGMT) and genes from the ABC transporter superfamily. The methyl groups from O-6-methylguanine, produced by alkylating agents such as TMZ, can be removed by MGMT permitting DNA repair of the toxic lesion. The most commonly used chemotherapeutic agents for the treatment of gliomas and GBM are lipophilic alkylating agents, allowing them to pass through the blood–brain barrier (BBB) [8]. Carmustine, lomustine, procarbazine, and temozolomide (TMZ) are DNA alkylating agents administered orally or intravenously for GBM treatment [9].

The superfamily of ABC transporters in humans is composed of 49 members distributed in seven subfamilies (ABCA–ABCG). Usually, these proteins are located in the cell membrane, but they can also be found in the membranes of the mitochondria, Golgi complex, and endoplasmic reticulum [10]. Many studies in the literature have shown that ABC transporters are associated with diseases including cystic fibrosis, Tangier’s disease, and cancer [11]. ABC transporters use ATP hydrolysis to actively transport a variety of compounds through the membrane including ions, sugars, amino acids, vitamins, lipids, hormones, and drugs, as well as larger molecules such as oligosaccharides [12,13].

The overexpression of P-glycoprotein (P-gp), encoded by the ABCB1 gene (or MDR1) and its drug efflux activity, is well known in GBM [6,14]. The overexpression of P-gp in GBM and many other tumors suggests that P-gp can be considered a marker of tumor aggressiveness [15].

In addition, other ABC transporters including ABCC1- 4 (MRP1- 4) confer resistance to a variety of chemotherapy drugs in many different types of cancer [16,17]. The use of P-gp inhibitors together with TMZ in U87MG and T98G GBM cells caused increased caspase-3 activity and induced apoptosis, while in an in vivo knockout model for ABCB1 (P-gp) the efficacy of TMZ treatment was higher than in control animals [18,19]. These studies demonstrate a clear relationship between increased P-gp protein expression and tumor resistance to TMZ.

Resistance to TMZ treatment is known to involve marked changes in miRNA expression and target mRNA gene expression, influencing GBM cell biology through diverse mechanisms including receptor tyrosine kinase pathways, autophagy, transcription factor activity, and DNA repair [5]. Some of these changes, such as altered miRNA expression and altered PI3K-Akt-NF-κB pathway activity, may be responsible for the reported changes in ABC transporter expression [20].

Alternatives to inactivate or downregulate ABC transporters in tumor cells include silencing through miRNAs and the combination of chemotherapeutic agents with specific bioactive nutrients such as curcumin derivatives or polyunsaturated fatty acids (PUFAs) [21,22,23,24].

There is evidence that PUFAs can interfere with resistance to chemotherapy in various tumors. It has been proposed that alterations in membrane phospholipid composition can affect membrane microdomains where ABC transporters are found, potentially altering ABC transporter activity. [25,26,27,28]. In breast cancer, PUFAs have been shown to increase oxidative stress and make the cells more susceptible to doxorubicin chemotherapy [29]. However, there is a paucity of studies testing the effects of PUFAs on GBM drug resistance [27,30,31].

Interestingly, TMZ has been shown to increase fatty acid uptake and oxidation by GBM cells, although these studies only used saturated palmitic acid (16:0) [7]. While increased saturated fatty acid uptake and oxidation may be beneficial to GBM cells in response to acute TMZ treatment, we hypothesized that the poorly oxidized PUFAs [32] would not have similar beneficial effects and could lead to increased cell death and reduced drug resistance. Previous studies have shown that the PUFAs GLA, DHA, and arachidonic acid (AA) can alter miRNA expression in the presence of acute TMZ exposure, although drug resistance was not considered in these earlier studies [33].

In the present study, we established a TMZ-resistant U87MG cell line for comparison with the TMZ-sensitive U87MG parental cell line. TMZ was chosen as it is the gold standard treatment for patients with GBMs containing methylated MGMT. This model was used to permit the comparison between the parent cell and the drug-resistant cell developed after long-term treatment, in preference to comparing cell lines with different genetic backgrounds such as U87MG and the drug-resistant T98G cell line. This follows a similar logic to the original studies of TMZ effects in U87MG cells [34].

These TMZ-resistant (TMZR) cells served as a model system representing the cells that survive and develop drug resistance after long-term TMZ treatment as used in the clinic post-surgery and post-radiotherapy [3]. These drug-resistant cells are a major causal factor in tumor recurrence in GBM patients [5]. These TMZR cells were developed to test the possible benefits of combination therapies using TMZ and the PUFAs (i) gamma-linolenic acid (GLA) (18:3, n-6), (ii) eicosapentaenoic acid (EPA) (20:5 n-3), or (iii) docosahexaenoic acid (DHA) (22:6, n-3) upon drug-resistant GBM cells and to compare the effects with those found in drug-sensitive GBM cells.

## 2. Materials and Methods

### 2.1. Cell Culture

U87MG human glioblastoma cells were cultured in DMEM (Dulbecco’s Modified Eagle Medium—Gibco Inc., Thermo Fisher Scientific, Grand Island, NY, USA) supplemented with 10% (*v*/*v*) FBS (fetal bovine serum—Thermo Fisher Scientific, Grand Island, NY, USA), 50 units/mL penicillin, 50 μg/mL streptomycin. The U87MG cells were originally obtained from the ATCC and were regularly checked for mycoplasma by Hoechst 33342 staining and PCR analysis. Cells were maintained at 37 °C in a humidified atmosphere with 5% CO_2_ in 25 or 75 cm^2^ flasks, until the desired confluency. Cells were washed with PBS and trypsinized (trypsin 0.025%/EDTA 0.02%) for further use in experiments.

### 2.2. Establishing the U87MG-TMZR Temozolomide Drug-Resistant Cell Line

Cells were grown in culture medium as detailed in Section 2.1, in 24-well plates at a cell density of 3 × 10^4^ cells/well. Cells were exposed to gradually increasing concentrations of temozolomide (TMZ) (Cayman Chemical, Ann Arbor, MI, USA), starting at 1 μM and reaching the previously published IC_50_ of 25 μM [35], with fresh medium and TMZ added every 48 h and weekly trypsinization of cells to expand the cultures. Similar IC_50_ values have been reported for these cells and other GBM cell lines [36].

The treatment scheme was as follows: 0–7 days, 1 μM; 8–14 days, 2 μM; 15–21 days, 4 μM; 22–28 days, 8 μM; 29–35 days, 10 μM; 36–45 days, 15 μM; 46–60 days, 20 μM; 61–90 days, 25 μM. After 90 days, cells were well adapted to 25 μM TMZ, stocks were frozen in DMEM/10%FBS/10% DMSO (Sigma Aldrich, São Paulo, Brazil) and stored in liquid nitrogen for use in all future experiments.

### 2.3. Determining the IC_50_ Values of Temozolomide for U87MG and U87MG-TMZR Cells

U87MG and U87MG-TMZR cells were seeded at 3 × 10^4^ cells/well in 24-well plates and after 24 h the cells were treated with TMZ at 0, 6.25, 12.5, 25, 50, 75, 100, 150, or 200 µM (Cayman Chemical, Ann Arbor, MI, USA). Culture medium and treatments were changed every 24 h. After 72 h cells were photographed, trypsinized, and cell counts were performed in a Neubauer chamber. All treatment concentrations were tested in quadruplicate. The IC_50_ was calculated using Graph Pad Prism 9 (GraphPad Prism 9 Software, San Diego, CA, USA).

### 2.4. Cell Counts Assay

Cells were seeded at 3 × 10^4^ cells/well in 24-well plates and after 24 h the cells were treated with gamma-linolenic acid, eicosapentaenoic acid or docosahexaenoic acid at 25, 50, 100, or 150 µM (Cayman Chemical, Ann Arbor, MI, USA) complexed with fatty-acid-free bovine serum albumin (ALB) (Sigma Aldrich, São Paulo, Brazil) as previously described [37]. ALB was used as the vehicle control (Sigma Aldrich, São Paulo, Brazil). Cells were concomitantly grown in the presence or absence of 25 μM TMZ. Culture medium and treatments were changed every 24 h. The cells and medium were collected at 24 h, 48 h, and 72 h and stained with 0.4% trypan blue to distinguish viable from unviable cells (Sigma Aldrich, São Paulo, Brazil). Cell counts were performed in a Neubauer chamber. All treatments were tested at least three times in triplicate.

### 2.5. Gas Chromatography-Mass Spectrometry Analysis

Cells were seeded at 3 × 10^4^ cells/well in 24-well plates and after 24 h the cells were treated with 100 µM gamma-linolenic acid, eicosapentaenoic acid or docosahexaenoic acid (Cayman Chemical, Ann Arbor, MI, USA) complexed with fatty-acid-free bovine serum albumin (ALB) (Sigma Aldrich, São Paulo, Brazil) as previously described [37]. ALB was used as the vehicle control (Sigma Aldrich, São Paulo, Brazil). Cells were concomitantly grown in the presence or absence of 25 µM TMZ. Culture medium and treatments were changed every 24 h. After 72 h, cells were collected by trypsinization, centrifuged at 250× *g* for 5 min, and washed with PBS, then the pellets were frozen and stored in liquid nitrogen. Lipids were extracted from cell pellets by the method of Folch [38] and fatty acid methyl esters were formed by the sulfuric acid/anhydrous methanol method [39].

The fatty acid methyl esters were separated on a DB-23 column ((50% cyanopropyl)methyl polysiloxane, 0.25 µm film thickness, 0.250 mm × 60 m), injection port 220 °C, column-mass spectrometer interface 250 °C, carrier gas helium (99.999%), in a Shimadzu GCMS model QP5050 (Shimadzu, Kyoto, Japan). The oven program used was as follows: 150 °C held for 2 min post injection, then 150–200 °C at a rate of 10 °C/min; 200–230 °C at 1.3 °C/min; 230–250 °C at 10 °C/min. Electron impact (EI)-MS analysis was in scan mode, mass range 35–500 *m*/*z*, scan interval 0.5 s. Samples were run in duplicate and individual fatty acid methyl esters were identified by comparison with authentic fatty acid standard retention times and mass spectra [40].

### 2.6. Cell Cycle Assay—Flow Cytometry

Cells were seeded at 9 × 10^4^ in a 12-well plate. After 24 h, the cells were treated with 100 µM gamma-linolenic acid, eicosapentaenoic acid, or docosahexaenoic acid (Cayman Chemical, Ann Arbor, MI, USA) complexed with fatty-acid-free bovine serum albumin (ALB) (Sigma Aldrich, Brazil) as previously described [37]. ALB was used as the vehicle control (Sigma Aldrich, Brazil). Cells were concomitantly grown in the presence or absence of 25 µM TMZ. Culture medium and treatments were changed every 24 h. After 72 h of treatment the cells were trypsinized, followed by centrifugation at 250× *g* for 5 min. The cell pellet was rinsed with ice-cold PBS, centrifuged, and resuspended in ice-cold 70% ethanol for at least 24 h. After ethanol fixation, cells were again washed with ice-cold PBS and incubated for 30 min with 500 μL of a staining solution (20 μg/μL propidium iodide, 50 μg/μL RNAse A and 0.1% Triton X-100). At the end of incubation, cells were centrifuged, resuspended in ice-cold PBS, and kept on ice before analysis. Cell cycle phase was determined by propidium iodide fluorescence detection of 10,000 events in a Guava^®^ easyCyte flow cytometer (Millipore, Burlington, MA, USA) using a λ_ext._ of 532 nm for propidium iodide detection [41,42].

### 2.7. ABC Transporter Activity Assay

Cells were grown in culture medium as detailed in Section 2.1, in 25 cm^2^ flasks in the presence of 100 µM gamma-linolenic acid, eicosapentaenoic acid, or docosahexaenoic acid (Cayman Chemical, Ann Arbor, MI, USA) complexed with fatty-acid-free bovine serum albumin (ALB) (Sigma Aldrich, São Paulo, Brazil) as previously described [37]. ALB was used as the vehicle control (Sigma Aldrich, São Paulo, Brazil). Cells were concomitantly grown in the presence or absence of 25μM TMZ. Culture medium and treatments were changed every 24 h.

After 72 h, cells were collected by trypsinization, centrifuged at 250× *g* for 5 min, washed with PBS, and the pellets resuspended in DMEM containing rhodamine 123 (R123) (Cayman Chemical, Ann Arbor, MI, USA). The cells were incubated at 37 °C in a humidified atmosphere with 5% CO_2_ for 60 min, then centrifuged and washed with PBS. The cells were placed in fresh DMEM and incubated at 37 °C in a humidified atmosphere with 5% CO_2_. At 20 min intervals aliquots were collected and cells were separated from the medium by centrifugation, washed with PBS, and resuspended in PBS. The medium and the resuspended cells were placed separately in a 96-well plate and fluorescence was determined at λ_ext._ 490 nm and λ_em._ 530 nm using a Synergy H1 Hybrid Multi-mode Reader (Biotek, Winooski, VT, USA).

### 2.8. Real Time qRT-PCR

The extraction of total RNA from previously treated cells was carried out with TRIzol reagent (Invitrogen, Carlsbad, CA, USA), followed by ethanol precipitation, as described by manufacturers. Double-strand cDNAs were synthesized using 2 µg of total RNA and M-MLV transcriptase (Invitrogen, Frederick, MD, USA) following the manufacturer’s instructions. Real-time quantitative reverse transcription PCR was carried out with Syber Green PCR Master Mix (Applied Biosystems, Warrington, UK) following the manufacturer’s instructions in a 7300 Real Time PCR System (Applied Biosystems, Waltham, MA, USA). TATA Box binding protein (TBP) expression was used as a housekeeping gene to normalize RNA expression, based on previous studies from the laboratory [40]. The cycle threshold (Ct) of each sample was determined, and the relative expression was calculated using 2^−ΔΔCt^ [43].

### 2.9. Primer Design

All primers used in this study were designed using PerlPrimer Software and the sequences deposited at the GenBank (www.ncbi.nlm.nih.gov) accessed on 6 January 2018 [44]. The primers used in this study were as follows (Gene: Forward; Reverse):
ABCB1: Forward: 5′-GAACCTGTATTGTTTGCCACC-3′Tm: 57.70 °C
Reverse: 5′-ACAGCTTTCTCAATCTCATCCA-3′Tm: 57.44 °CABCC1: Forward: 5′-AGGAGAGATCATCATCGATGG-3′Tm: 56.48 °C
Reverse: 5′-GCCTTCTGCACATTCATGG-3′Tm: 56.97 °CABCC3: Forward: 5′-CCTGTATGTGGGTCAAAGTGCG-3′Tm: 61.76 °C
Reverse: 5′-CCCAGCCTCAGGGAAGTGTTG-3′Tm: 62.94 °CABCC4: Forward: 5′-CCATTGAAGATCTTCCTGG-3′Tm: 52.67 °C
Reverse: 5′-GGTGTTCAATCTGTGTGC-3′Tm: 54.01 °CTBP: Forward: 5′-CCACTCCACTGTATCCCTCC-3′Tm: 58.88 °C
Reverse: 5′-GACTGTTCTTCACTCTTGGCT-3′Tm: 57.88 °C

### 2.10. Western Blot

Cells were cultivated in the presence of 100 µM GLA, EPA, DHA (Cayman Chemical, Ann Arbor, MI, USA), or fatty-acid-free albumin control (Sigma-Aldrich, Brazil) for 72 h. Cells were trypsinized, pelleted by centrifugation at 4 °C for 3 min at 410× *g*, and washed with cold PBS 3× before snap-freezing the samples in liquid nitrogen and storing at −80 °C until the time of extraction. The frozen pellet was thawed on ice, lysis buffer containing a protease inhibitor mix (cOmplete protease inhibitor, Roche, IN, USA) was added, and the pellet was re-suspended for 5 min and left on ice for 30 min. After centrifugation at 9520× *g* at 4 °C for 10 min, the protein content of the sample of interest was determined against a protein concentration curve of bovine serum albumin. Laemmli sample buffer was added (1:1) and the samples were heated (95 °C) for 5 min. The total protein extracts were frozen at −80 °C for long-term storage.

An amount of 40 μg of protein from each sample was loaded onto a 4% SDS-PAGE stacking gel with a 10% separation gel and after electrophoretic separation (75 V stacking gel/100 V separation gel) the samples were transferred to nitrocellulose membranes (Thermo Scientific, Rockford, IL, USA). The transfer was confirmed by staining the membrane with 0.5% Ponceau red solution (Sigma-Aldrich, São Paulo, Brazil).

Immunoblotting followed standard methods which were as follows: The washed membranes were blocked with 5% (*w/v*) fat-free milk for 1 h and after removal of excess blocking solution by washing, the membranes were incubated overnight with the primary antibodies (ABCC1 [1:100]; ABCC4 [1:500]; beta-actin [1:1000], all Abcam, MA, USA). The membranes were washed and incubated with secondary antibodies for 2 h at room temperature (anti-mouse-HRP (ABCC1), anti-goat-HRP (ABCC4), anti-mouse-HRP (beta-actin) [1:1000], all Abcam, USA). Finally, the membranes were washed and developed with enhanced chemiluminescence (Clarity Western ECL substrate—Bio-Rad Laboratories Inc., Brazil) in a Syngene G-BOX using GeneSys software. The ImageJ program version 1.53t was used to determine relative band intensities.

### 2.11. Nile Red Staining of Neutral Lipids

Cells were seeded at 3 × 10^4^ cells/well in 24-well plates on glass coverslips and after 24 h the cells were treated with 100 µM gamma-linolenic acid, eicosapentaenoic acid or docosahexaenoic acid (Cayman Chemical, Ann Arbor, MI, USA) complexed with fatty-acid-free bovine serum albumin (ALB) (Sigma Aldrich, São Paulo, Brazil) as previously described [37]. ALB was used as the vehicle control (Sigma Aldrich, São Paulo, Brazil). After 72 h the cells were fixed in 4% formaldehyde freshly prepared from paraformaldehyde, in 0.1 M potassium phosphate, pH 7.2. The cells were stained with 1 mg/mL Nile Red and 1 mg/mL DAPI. The coverslips were mounted in Vectashield (Vector Laboratories, Newark, CA, USA) on glass slides and viewed on a Nikon Optiphot-II epifluorescence microscope equipped with a Cool Snap Pro camera and Image Pro Plus software as previously described [45,46].

### 2.12. Statistical Analysis

All data were plotted and analysed using GraphPad Prism 9 (GraphPad Prism 9 Software, San Diego, CA, USA). Values express the arithmetic mean ± standard error. Analysis between two groups was performed with Student’s *t*-test. Analysis performed between three or more groups, considering only one variable was performed with One-way ANOVA followed by Dunnett’s test. Analysis between three or more groups, considering two variables were performed with Two-way ANOVA followed by Bonferroni test. The differences were considered statistically significant at *p* < 0.05.

## 3. Results

**Figure 1:**
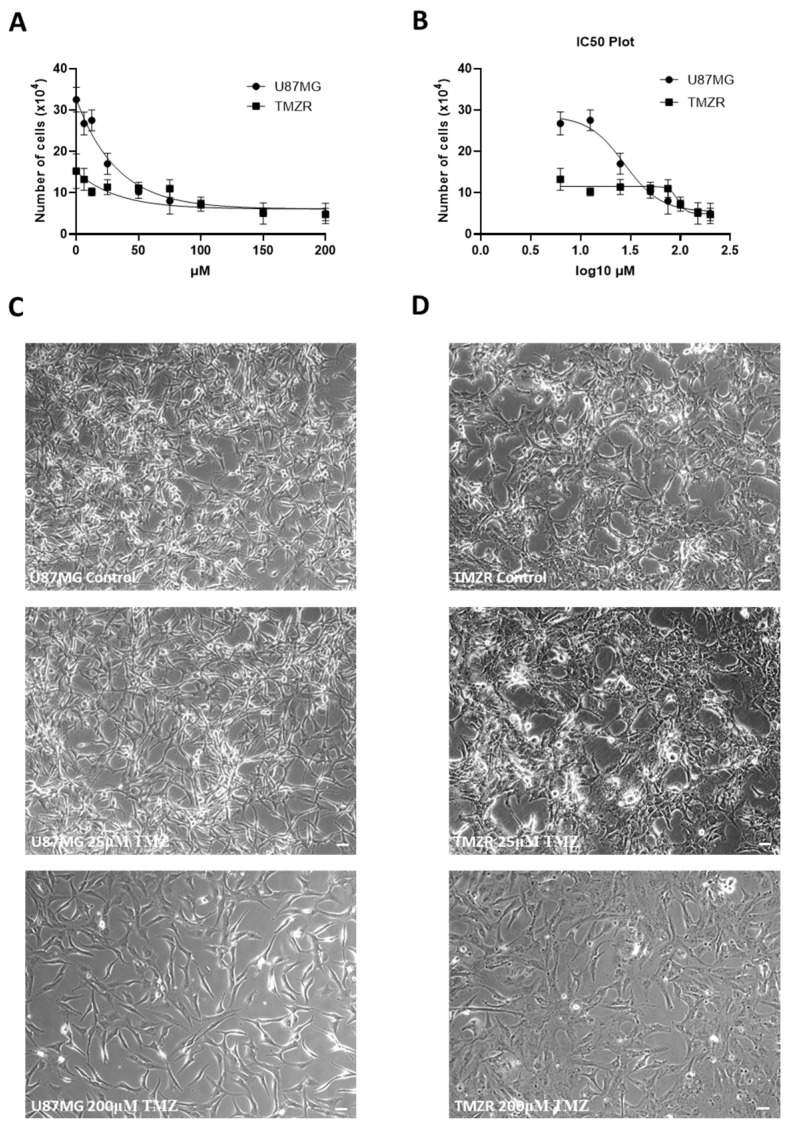
Development of TMZ resistance in U87MG cells: (**A**) Concentration curve of cell number after 72 h of exposure to varying concentrations of TMZ for U87MG and TMZ-resistant cells (TMZR); (**B**) IC_50_ plot for U87MG and TMZR cells—U87MG IC50 = 23.6 µM; TMZR IC50 = 94.3 µM. (**C**) Phase contrast images of U87MG cells at 0, 25 and 200 µM TMZ. (**D**) Phase contrast images of TMZR cells at 0, 25 and 200 µM TMZ. Data are presented as mean ± SEM, N = 4. Scale bar in (**C**,**D**) = 50 µm.

In Figure 1A, the dose-dependent inhibitory effects of TMZ can be seen on U87MG cell number after 72 h and in Figure 1B the calculated IC_50_ for TMZ was 23.6 µM. In Figure 1A, the TMZ-resistant (TMZR) cells did not show the same dose-dependent inhibition with TMZ as seen for the U87MG cells, and in Figure 1B the calculated IC_50_ for TMZ was much higher at 94.3 µM. In Figure 1C,D, increasing concentrations of TMZ are seen to reduce cell number and alter morphology at 200 µM.

These data prove that drug resistance was acquired by long-term exposure of the cells to TMZ at increasing doses as described in the protocol in Section 2.2. Further experiments in the study were carried out using 25 µM TMZ, close to the IC_50_ of U87MG cells and a concentration at which the TMZR cells’ growth was unaffected. At 25 µM TMZ, the final concentration of the DMSO vehicle was 0.25% and did not interfere with cell growth, e.g., TMZR = 11.4 ± 0.9 × 10^4^ vs. TMZR DMSO cell number = 14.5 ± 1.3 × 10^4^). This concentration was used to avoid causing excessive cell death in the TMZ-sensitive U87MG parent cells.

**Figure 2:**
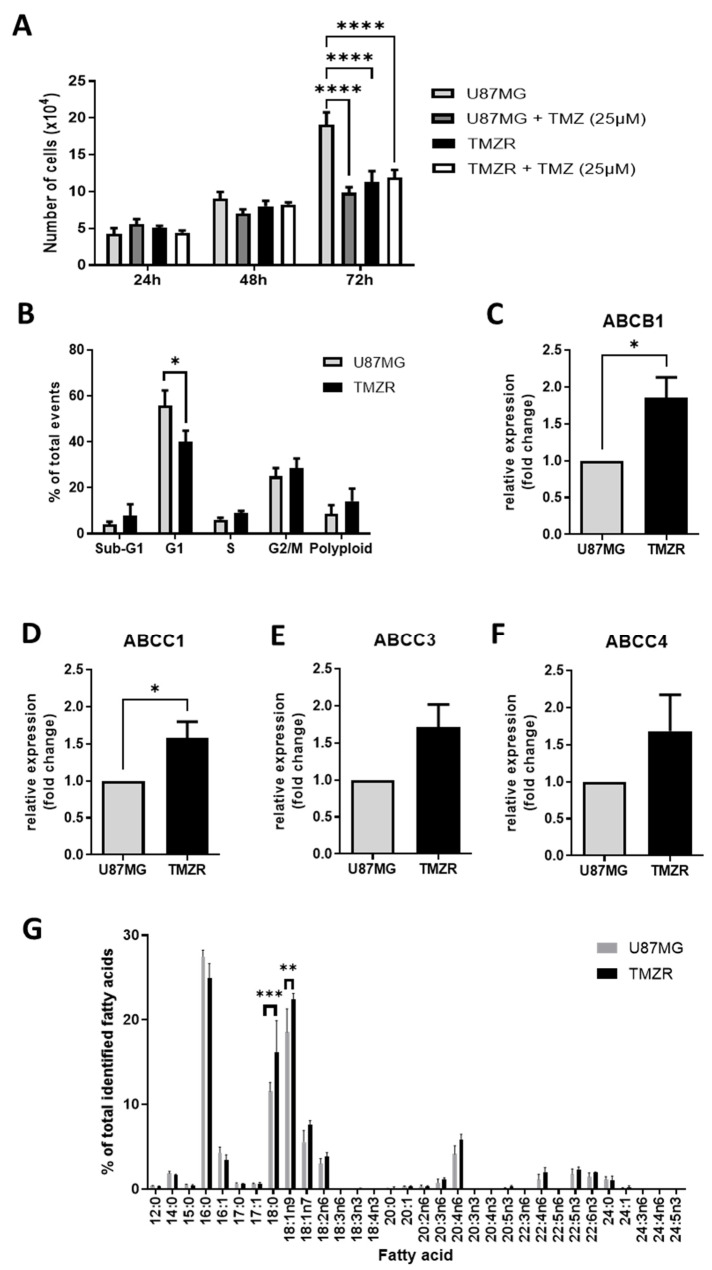
Effects of the development of TMZ resistance on U87MG cells: (**A**) 25 µM TMZ in U87MG and TMZR cells; (**B**) flow cytometer analysis of cell cycle; (**C**) qRT-PCR of ABCB1 expression; (**D**) qRT-PCR of ABCC1 expression; (**E**) qRT-PCR of ABCC3 expression; (**F**) qRT-PCR of ABCC4 expression; (**G**) GCMS analysis of fatty acid composition. Data are presented as mean ± SEM, N = 3. Differences were considered significant at *p* < 0.05. * = *p* < 0.05; ** = *p* < 0.01; *** = *p* < 0.001; **** = *p* < 0.0001.

In Figure 2A, the inhibitory effects of 25 µM TMZ can be seen on U87MG cell growth (number of cells) at 48 and 72 h. This inhibitory effect was not observed for the TMZR cells at any time point (Figure 2A). The TMZR cells had a significantly reduced G1 phase of the cell cycle (Figure 2B). The mRNA expression of ABCB1 and ABCC1 was significantly increased in the TMZR cells after TMZ resistance was established (Figure 2C–F). The fatty acid composition of the TMZR cells was significantly altered in comparison with U87MG cells, with an increase in 18:0 and 18:1 n-9 content (Figure 2G).

**Figure 3:**
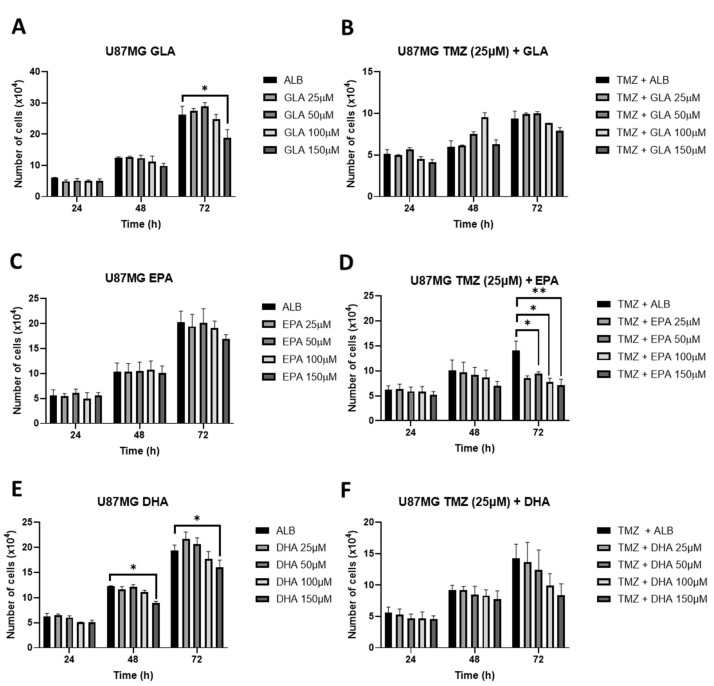
Effects of gamma-linolenic acid (GLA), eicosapentaenoic acid (EPA), and docosahexaenoic acid (DHA) on U87MG cell counts: (**A**,**C**,**E**) Graphs show the results after 24, 48, or 72 h of treatment with PUFAs. (**B**,**D**,**F**) Graphs show the results after 24, 48 or 72 h of treatment with PUFAs in the presence of TMZ. Data are presented as mean ± SEM, N = 3. Differences were considered significant at *p* < 0.05. * = *p* < 0.05; ** = *p* < 0.01.

The effects of different concentrations of GLA, EPA, or DHA on U87MG cell counts are presented in Figure 3. In U87MG cells, GLA decreased cell counts at 150 µM after 72 h, while in the presence of TMZ this effect was not observed (Figure 3A,B). The presence of EPA did not alter U87MG cell counts in the absence of TMZ but showed a significant reduction in cell counts at 50, 100, and 150 µM after 72 h in the presence of TMZ (Figure 3C,D). In U87MG cells, DHA caused a significant decrease in cell counts at 150 µM after 48 and 72 h (Figure 3E). A tendency to decrease cell counts was seen in the presence of TMZ at 72 h but did not reach statistical significance (Figure 3F).

**Figure 4:**
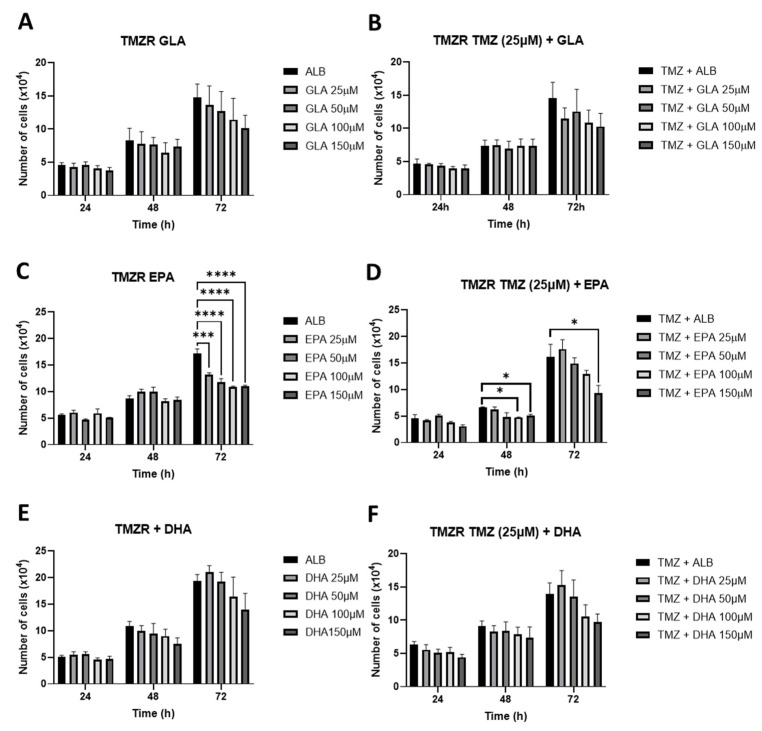
Effects of gamma-linolenic acid (GLA), eicosapentaenoic acid (EPA), and docosahexaenoic acid (DHA) on TMZR cell counts: (**A**,**C**,**E**) Graphs show the results after 24, 48, or 72 h of treatment with PUFAs. (**B**,**D**,**F**) Graphs show the results after 24, 48, or 72 h of treatment with PUFAs in the presence of TMZ. Data are presented as mean ± SEM, N = 3. Differences were considered significant at *p* < 0.05. * = *p* < 0.05; *** = *p* < 0.001; **** = *p* < 0.0001.

The effects of different concentrations of GLA, EPA, or DHA on TMZR cell counts are presented in Figure 4. In TMZR cells, GLA showed a tendency to decrease cell counts at 150 µM after 72 h in the absence and presence of TMZ but did not reach statistical significance (Figure 4A,B). In contrast, EPA caused a significant decrease in cell counts in TMZR cells at 25, 50, 100, and 150 µM after 72 h (Figure 4C). In the TMZR cells a decrease in cell counts was seen at 100 and 150 µM EPA after 48 h and at 150 µM after 72 h in the presence of TMZ (Figure 4D). In a similar manner to GLA, in TMZR cells DHA showed a tendency to decrease cell counts at 150 µM after 72 h in the absence and presence of TMZ but did not reach statistical significance (Figure 4E,F).

**Figure 5:**
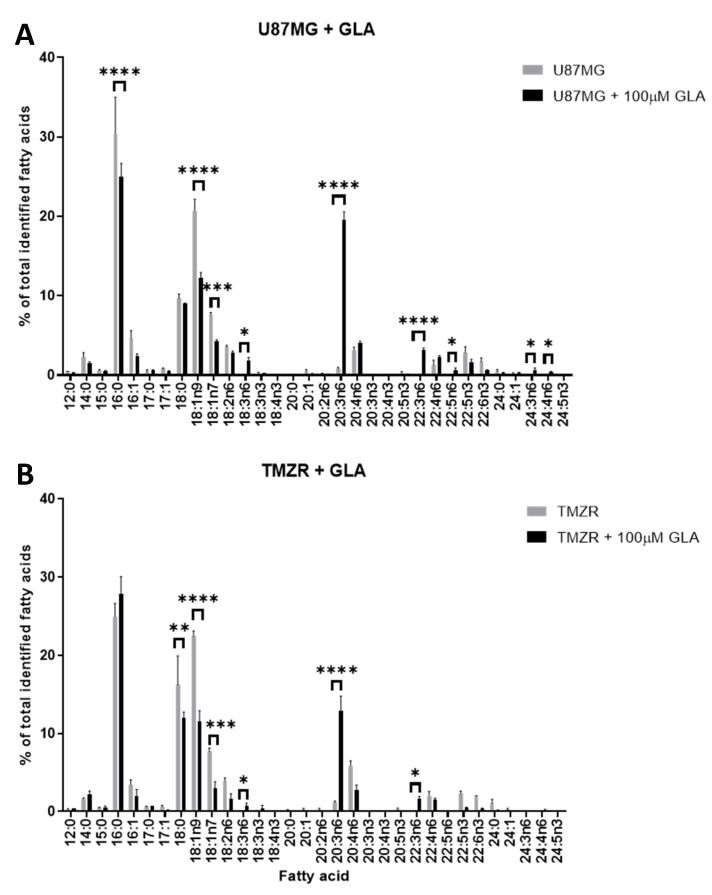
Effects of gamma-linolenic acid (GLA) on fatty acid composition of U87MG and TMZR cells (**A**) U87MG cells (**B**) TMZR cells. Data are presented as mean ± SEM, N = 3. Differences were considered significant at *p* < 0.05. * = *p* < 0.05; ** = *p* < 0.01; *** = *p* < 0.001; **** = *p* < 0.0001.

The effects of 100 µM GLA on the fatty acid composition of U87MG cells and TMZR cells are presented in Figure 5. U87MG cells had a significant decrease in 16:0, 18:1 n-9, and 18:1 n-7, and significant increases in 18:3 n-6, 20:3 n-6, 22:3 n-6, 22:5 n-6, 24:3 n-6, and 24:4 n-6 (Figure 5A). TMZR cells had a significant decrease in 18:0, 18:1 n-9, and 18:1 n-7, and significant increases in 18:3 n-6, 20:3 n-6, and 22:3 n-6 (Figure 5B).

**Figure 6:**
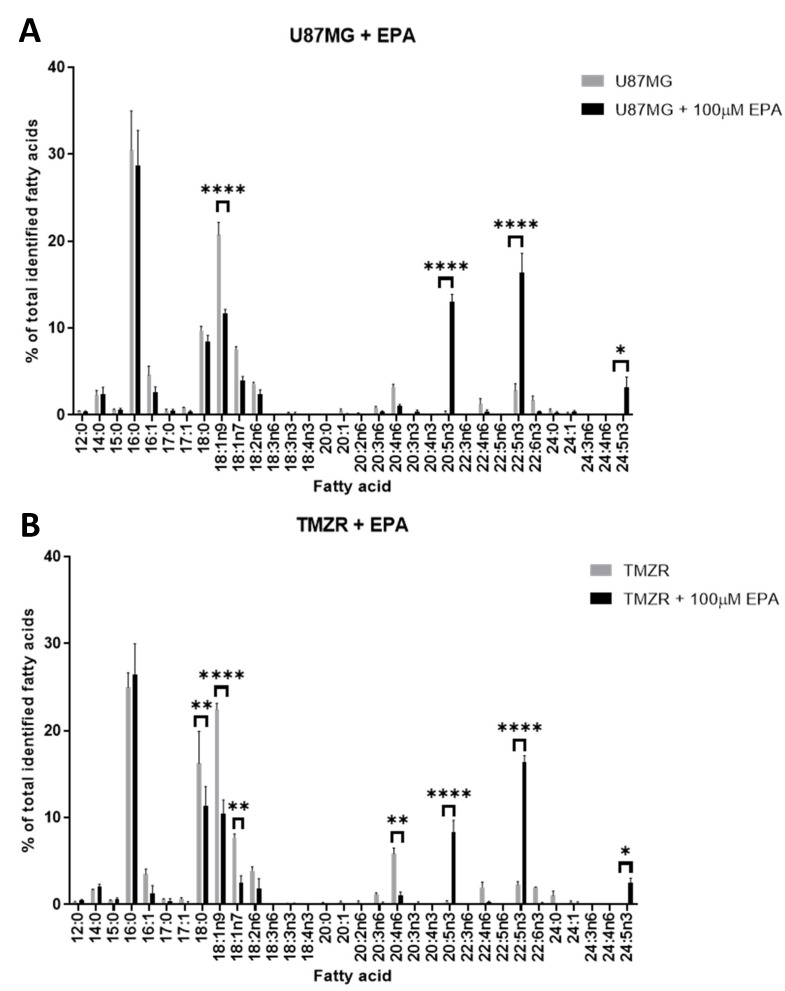
Effects of eicosapentaenoic acid (EPA) on fatty acid composition of U87MG and TMZR cells (**A**) U87MG cells (**B**) TMZR cells. Data are presented as mean ± SEM, N = 3. Differences were considered significant at *p* < 0.05. * = *p* < 0.05; ** = *p* < 0.01; **** = *p* < 0.0001.

The effects of 100 µM EPA on the fatty acid composition of U87MG cells and TMZR cells are presented in Figure 6. U87MG cells had a significant decrease in 18:1 n-9 and significant increases in 20:5 n-3, 22:5 n-3, and 24:5 n-3 (Figure 6A). TMZR cells had a significant decrease in 18:0, 18:1 n-9, 18:1 n-7, and 20:4 n-3 and significant increases in 20:5 n-3, 22:5 n-3, and 24:5 n-3 (Figure 6B).

**Figure 7:**
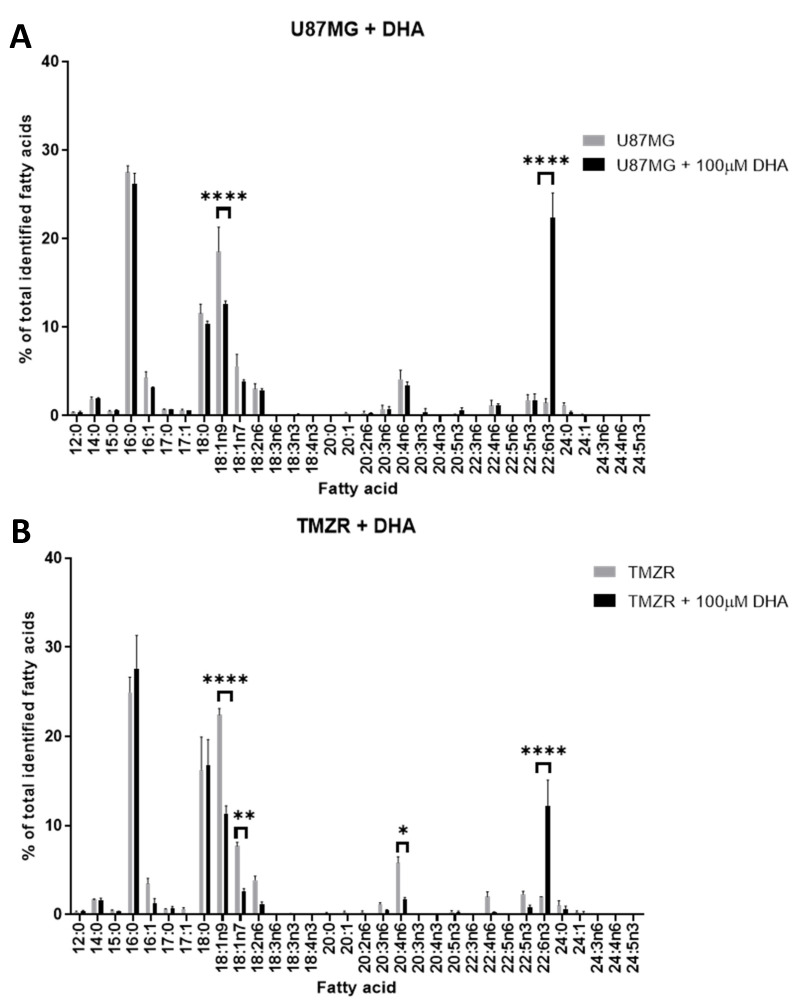
Effects of docosahexaenoic acid (DHA) on fatty acid composition of U87MG and TMZR cells (**A**) U87MG cells (**B**) TMZR cells. Data are presented as mean ± SEM, N = 3. Differences were considered significant at *p* < 0.05. * = *p* < 0.05; ** = *p* < 0.01; **** = *p* < 0.0001.

The effects of 100 µM DHA on the fatty acid composition of U87MG cells and TMZR cells are presented in Figure 7. U87MG cells had a significant decrease in 18:1 n-9 and a significant increase in 22:6 n-3 (Figure 7A). TMZR cells had a significant decrease in 18:1 n-9, 18:1 n-7, and 20:4 n-6 and a significant increase in 22:6 n-3 (Figure 7B).

Overall, PUFAs caused a reduction in saturated and monounsaturated fatty acids with an accumulation of PUFAs. Both GLA and EPA were readily elongated and GLA was also desaturated, while DHA was incorporated without evidence for potential retroconversion to EPA.

**Figure 8:**
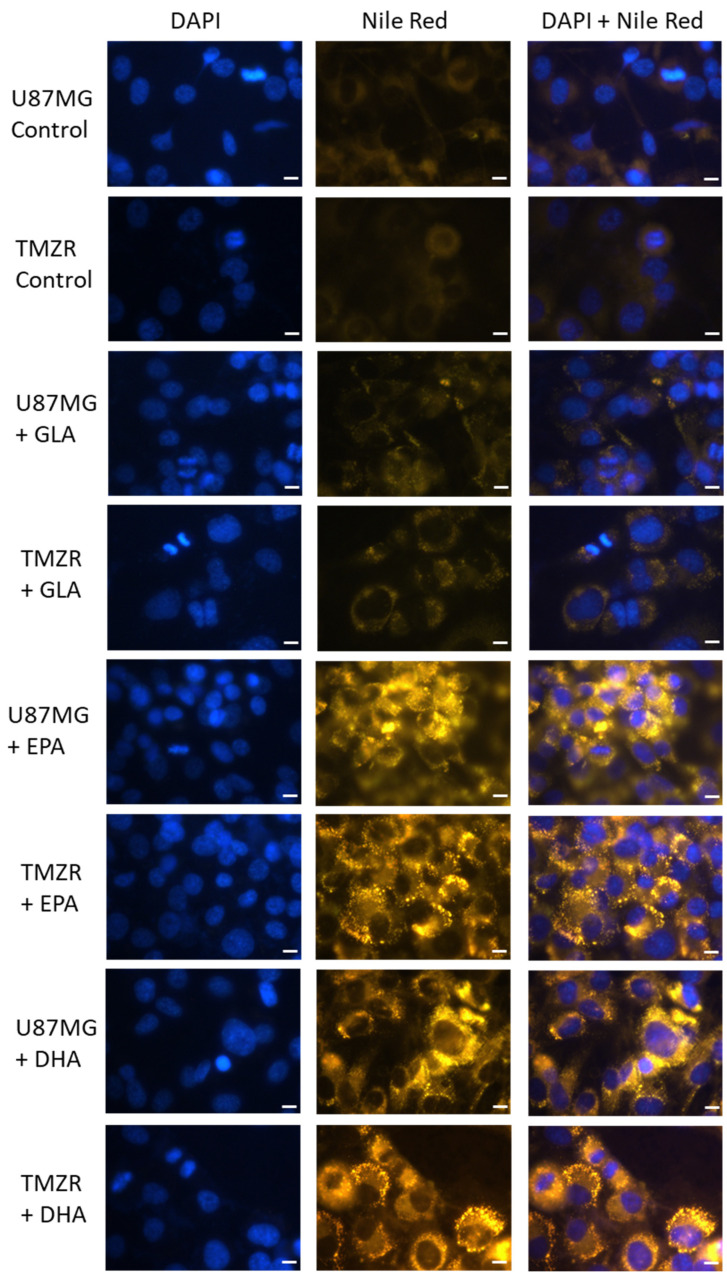
Effects of gamma-linolenic acid (GLA), eicosapentaenoic acid (EPA), and docosahexaenoic acid (DHA) on Nile Red staining of U87MG and TMZR cells. DAPI staining in blue, Nile Red in yellow. The control cells were exposed to a fatty-acid-free albumin vehicle at the same concentration as the fatty-acid-treated cells. Scale bar = 10 µm.

The fluorescent neutral lipid stain Nile Red was used to show the accumulation of lipid droplets in U87MG and TMZR cells treated with 100 µM GLA, EPA, or DHA for 72 h and DAPI was used as a nuclear stain (Figure 8). The morphology of GLA-treated cells showed an accumulation of lipid droplets in the cytoplasm as previously described in glioma cells [37,45,46]. The morphology of EPA-treated cells also showed an accumulation of lipid droplets in the cytoplasm as previously described in tumor cells [47]. Finally, the morphology of DHA-treated cells showed an accumulation of lipid droplets in the cytoplasm. In comparison, the U87MG and TMZR cells exposed only to the fatty-acid-free albumin vehicle showed sparse lipid droplets in the cytoplasm (Figure 8).

While the accumulation of lipid droplets in the cytoplasm can have a protective role under certain conditions, excessive accumulation can lead to the eventual induction of cell death as previously described in other tumors [37,47,48]. Using stimulated Raman scattering (SRS) microscopy, U87MG cells were seen to accumulate saturated (palmitic acid), monounsaturated (oleic acid), and polyunsaturated fatty acids (EPA). EPA was found to cause the highest lipotoxicity although the concentrations used were higher than in the present study [48].

**Figure 9:**
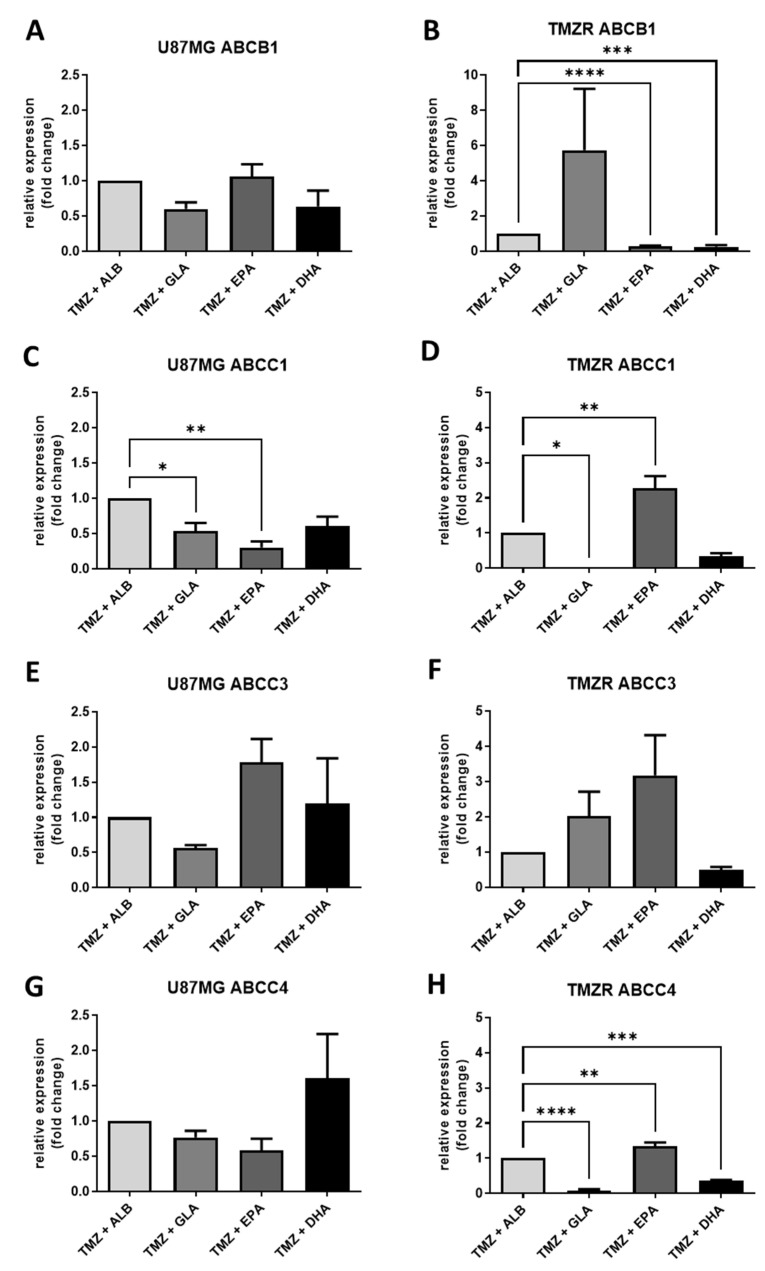
Effects of gamma-linolenic acid (GLA), eicosapentaenoic acid (EPA), and docosahexaenoic acid (DHA) on mRNA expression of ABC transporters. (**A**,**C**,**E**,**G**) ABCB1, ABCC1, ABCC3, and ABCC4 in U87MG cells; (**B**,**D**,**F**,**H**) ABCB1, ABCC1, ABCC3, and ABCC4 in TMZR cells;. Data are presented as mean ± SEM, N = 3. Differences were considered significant at *p* < 0.05. * = *p* < 0.05; ** = *p* < 0.01; *** = *p* < 0.001; **** = *p* < 0.0001.

The presence of 100 µM GLA, EPA, or DHA caused significant changes in ABC transporter mRNA expression as shown in Figure 9. In U87MG cells, GLA caused a significant decrease in ABCC1 (Figure 9C). In TMZR cells, GLA caused a significant decrease in ABCC1 and ABCC4 expression (Figure 9D,H). EPA caused a significant decrease in ABCC1 in U87MG cells (Figure 9C,G). However, in TMZR cells EPA caused a significant decrease in ABCB1 and a significant increase in ABCC1 and ABCC4 (Figure 9B,D,H). In U87MG cells, DHA had no significant effect on ABC transporter expression (Figure 9A,C,E,G). DHA caused a significant decrease in ABCB1 and ABCC4 expression in TMZR cells (Figure 9B,H).

**Figure 10:**
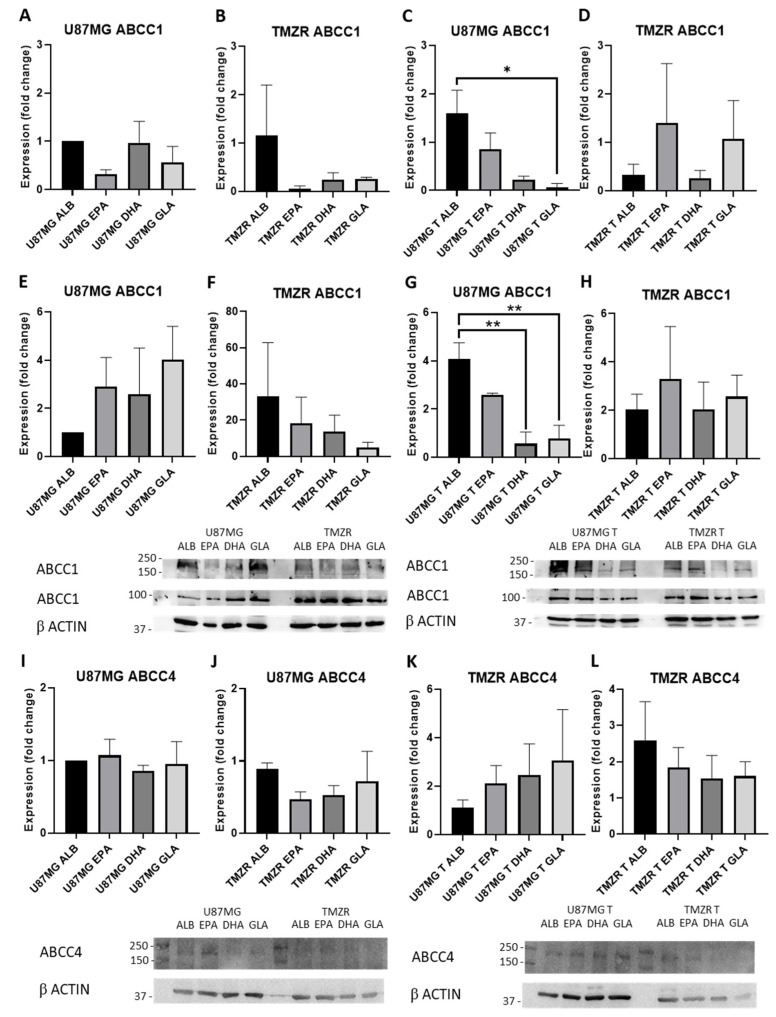
Effects of gamma-linolenic acid (GLA), eicosapentaenoic acid (EPA), and docosahexaenoic acid (DHA) on protein expression of ABCC1 and ABCC4 transporters: (**A**–**D**) ABCC1 190 KDa band in (**A**) U87MG; (**B**) TMZR; (**C**) U87MG + 25 µM TMZ; (**D**) TMZR + 25 µM TMZ; (**A**–**D**) normalized against U87MG ALB expression. (**E**–**H**) ABCC1 100 KDa band in (**E**) U87MG; (**F**) TMZR; (**G**) U87MG + 25 µM TMZ; (**H**) TMZR + 25 µM TMZ; (**E**–**H**) normalized against U87MG ALB expression (**I**–**L**) ABCC4 170 KDa band in (**I**) U87MG; (**J**) TMZR; (**K**) U87MG + 25 µM TMZ; (**L**) TMZR + 25 µM; (**I**–**L**) normalized against U87MG ALB expression. Data are presented as mean ± SEM, N = 3. Differences were considered significant at *p* < 0.05. * = *p* < 0.05; ** = *p* < 0.01.

The presence of 100 µM GLA, EPA, or DHA caused significant changes in ABC transporter protein expression as shown in Figure 10. In U87MG + 25 µM TMZ, GLA caused a significant decrease in ABCC1 (Figure 10C). DHA also caused a significant decrease in ABCC1 expression, while EPA showed a tendency to decrease expression. In contrast, none of the fatty acids caused significant changes in ABCC4 expression.

**Figure 11:**
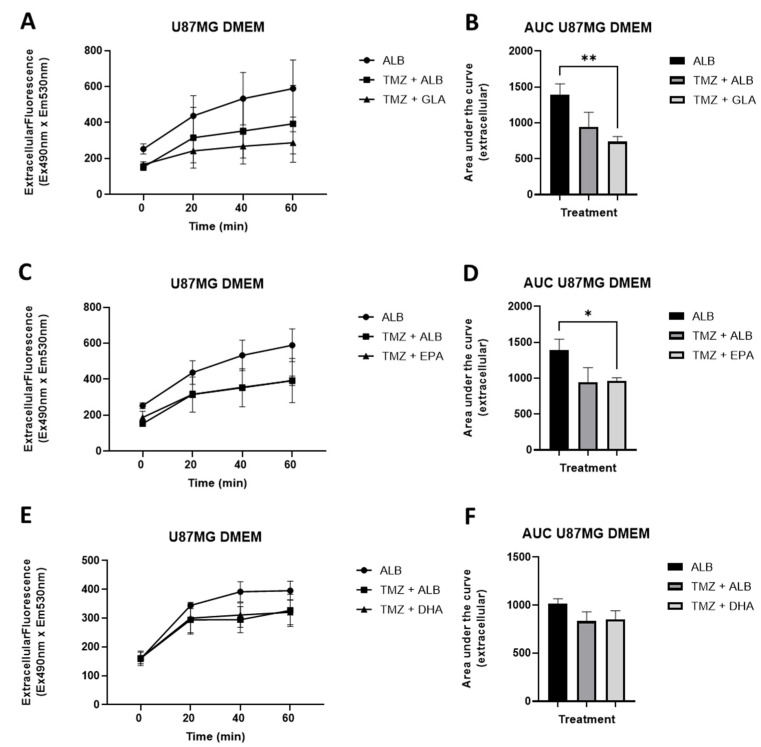
Effects of gamma-linolenic acid (GLA), eicosapentaenoic acid (EPA), and docosahexaenoic acid (DHA) on rhodamine 123 efflux in U87MG cells: (**A**) Efflux ± GLA. (**B**) Area under curve for efflux ± GLA. (**C**) Efflux ± EPA (**D**) Area under curve for efflux ± EPA. (**E**) Efflux ± DHA. (**F**) Area under curve for efflux ± DHA. Data are presented as mean ± SEM, N = 3. Differences were considered significant at *p* < 0.05. * = *p* < 0.05; ** = *p* < 0.01.

In the ABC transporter activity assay 100 µM GLA + TMZ caused a significant decrease in extracellular concentration of rhodamine 123 (R123) in comparison with the albumin control for U87MG cells. However, the presence of GLA did not alter the extracellular concentration of R123 in comparison with albumin + TMZ (Figure 11A,B).

**Figure 12:**
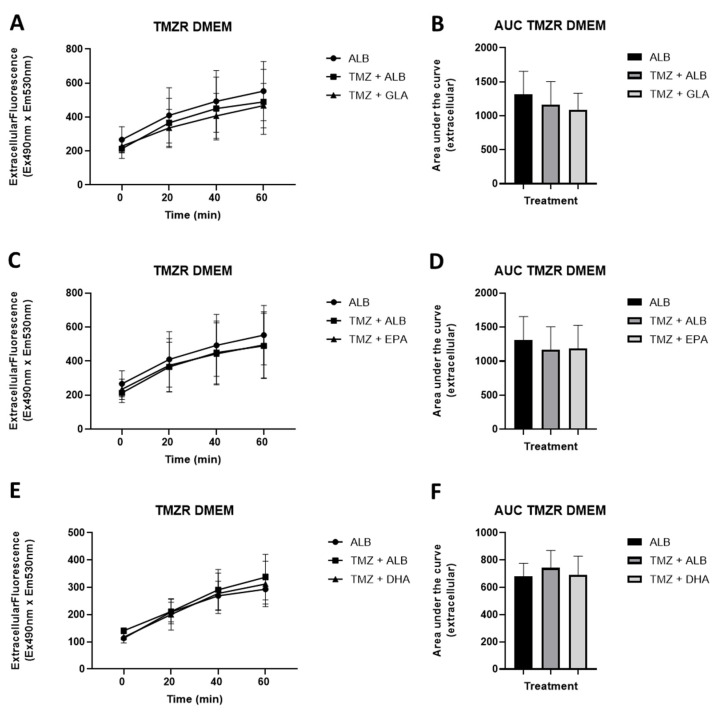
Effects of gamma-linolenic acid (GLA), eicosapentaenoic acid (EPA), and docosahexaenoic acid (DHA) on rhodamine 123 efflux in TMZR cells: (**A**) Efflux ± GLA. (**B**) Area under curve for efflux ± GLA. (**C**) Efflux ± EPA. (**D**) Area under curve for efflux ± EPA. (**E**) Efflux ± DHA. (**F**) Area under curve for efflux ± DHA. Data are presented as mean ± SEM, N = 3. Differences were considered significant at *p* < 0.05.

In U87MG cells, 100 µM EPA + TMZ caused a significant decrease in extracellular concentration of R123 in comparison with the albumin control. However, the presence of EPA did not alter the extracellular concentration of R123 in comparison with albumin + TMZ for U87MG cells (Figure 11C,D). The presence of DHA had no effect on U87MG extracellular R123 concentration (Figure 11E,F). In contrast with the data for U87MG cells, the TMZR cells showed no significant changes in extracellular R123 in the presence of 100 µM GLA, EPA, or DHA of the PUFAs studied (Figure 12A–F).

## 4. Discussion

The TMZ-resistant cells developed in this study had a significantly increased IC_50_ for TMZ in comparison with the parent U87MG cells (94.3 vs. 23.6 µM). This was accompanied by a significant increase in the mRNA expression of ABCB1 and ABCC1 ABC transporters. TMZ is considered a substrate for the ABCB1 transporter [18,19]. Of particular interest was the significant change in fatty acid composition of the TMZR cells in response to TMZ. The cells had significantly more saturated fatty acids (18:0) than U87MG cells despite the same fatty acids being available in the culture medium. This suggests that a more saturated fatty acid composition is beneficial to the cells in the development of drug resistance. Indeed, studies have reported that P-gp activity (ABCB1) is stimulated by a saturated fatty acid environment and inhibited by an increase in PUFAs [27]. These changes in gene expression and fatty acid composition were accompanied by changes in the morphology of the TMZR cells when compared with the U87MG cells.

The most significant changes In cell counts were seen for the U87MG cells after 72 h in the presence of 50–150 µM EPA and TMZ. Significant decreases in cell counts were also seen in TMZR cells after treatment with EPA in both the absence and presence of TMZ. These data indicate that among the PUFAs GLA, EPA, and DHA, EPA is the most effective in inhibiting cell growth in both U87MG cells and more interestingly in TMZR cells. EPA was found to be the most lipotoxic fatty acid among palmitic acid, oleic acid, and EPA, in U87MG cells studied by stimulated Raman scattering (SRS) microscopy, although the concentrations used were higher than in the present study [48].

The analysis of the fatty acid composition of PUFA-treated cells showed considerable changes in composition depending upon the fatty acid used. GLA was readily incorporated, elongated, and desaturated up to 24:4 n6 in U87MG cells. These findings are similar to those seen with GLA in C6 rat glioma cells [46]. The presence of GLA in TMZR cells caused a significant drop in 18:0 content which was not seen in U87MG cells. EPA was also readily incorporated and elongated up to 24:5 n-3 in U87MG cells. These findings are similar to those seen with EPA in C6 rat glioma cells [46]. The presence of EPA also caused a significant decrease in 18:0 content in the TMZR cells but not in the U87MG cells. Finally, DHA was also incorporated into U87MG and TMZR cells but remained as DHA with no signs of its potential retroconversion to 22:5 n3 or EPA.

The significant inhibitory effects of GLA and EPA on mRNA expression of ABCC1 in U87MG supports the proposal that PUFAs can modulate ABC transporters. Previous studies have also shown the modulation of ABCB1 and ABCC1 by PUFAs in colon cancer [26]. In U87MG, GLA and DHA caused a significant decrease in ABCC1 protein expression, while EPA showed a tendency to decrease expression. In TMZR cells, GLA inhibited mRNA expression of ABCC1 and ABCC4 while DHA inhibited ABCB1 and ABCC4 expression. Interestingly, EPA had differing effects on individual ABC transporters in TMZR cells, inhibiting ABCB1 expression while increasing ABCC1 and ABCC4 expression. However, these effects were not seen for ABCC1 protein expression. In addition, none of the fatty acids caused significant changes in ABCC4 protein expression.

The drug efflux study using the fluorescent probe rhodamine 123 showed that either GLA or EPA in combination with TMZ caused significant reductions in R123 efflux from U87MG cells. However, these effects were not evident in the TMZR cells treated with any of the PUFAs. Further studies are required with additional substrates to identify whether PUFAs alter efflux activity for these or other ABC transporters.

Overall, these findings suggest that EPA may be an interesting option for combination with TMZ as it is readily incorporated into glioma cells where it alters the fatty acid composition, cell growth, morphology, ABC transporter gene expression, and efflux activity. It is particularly relevant that EPA had more striking effects on cells than DHA when we consider that the brain microenvironment where gliomas develop is rich in DHA while the levels of EPA are often undetectable. Indeed, studies in our laboratory have shown that EPA and its eicosanoid derivatives are almost undetectable in human glioma tissues in comparison with DHA and its docosanoid derivatives when analyzed by GCMS and LC-ESI-MS/MS [40].

This proof-of-principle study has shown the potential of PUFAs, and in particular EPA, to alter multiple drug resistance in GBM cells. However, it is limited by the use of only one parental cell line and one TMZ-resistant cell line. Clearly, future studies require the use of additional cell lines as well as primary cell cultures. In addition, the use of 3D spheroid cultures will allow testing of PUFA effects in more tumor-like culture conditions before taking these findings to in vivo studies in human GBM xenograft models.

## Data Availability

Access to original data can be provided upon reasonable request.
